# Effect of Diabetes Education on Complications and Diabetic Control Amongst Adult Patients with Diabetes in Madinah, Saudi Arabia

**DOI:** 10.3390/healthcare12171708

**Published:** 2024-08-26

**Authors:** Omar M. Al-Nozha, Ghazi H. Mogharbel, Ahmad S. Badawi, Abdulaziz K. Alawfi, Mohammed W. Aljayyar, Osamah N. Makhdoom, Husain M. Kateb, Anwar A. Sayed

**Affiliations:** 1 Department of Medicine, College of Medicine, Taibah University, Madinah 423535, Saudi Arabia; 2 College of Medicine, Taibah University, Madinah 423535, Saudi Arabia; 3 Department of Basic Medical Sciences, College of Medicine, Taibah University, Madinah 423535, Saudi Arabia

**Keywords:** diabetes complications, diabetes education, diabetes knowledge test, glycated hemoglobin, patient health education, type 1 diabetes, type 2 diabetes

## Abstract

Background: Diabetes is a chronic condition that may become dangerous if there is insufficient insulin to help the body function properly. The proper care for diabetes depends on how well patients observe guidelines and prescriptions; consequently, patient education is critical. Poor learning may cause bad treatment and complications or other problems related to the disease. Objectives: This study aims to evaluate patients’ knowledge of diabetes, assigning a knowledge (K) score out of 100, and investigate the possible impact of educating patients, through general means or via healthcare professionals, on patient knowledge of diabetes control demonstrated in the absence/presence of diabetic complications. Methods: This multi-center interview-based cross-sectional study used a questionnaire in Madinah, Saudi Arabia. This study was conducted on adults with diabetes who were aged 15–80. We used the Michigan Diabetic Knowledge Test (MDKT) to assess the knowledge of patients with diabetes. Results: This study included 364 participants. The gender distribution was 48.33% male and 51.67% female. Most of them had type 2 diabetes (T2DM) without insulin (48.63%), followed by those with T2DM on insulin (36.26%), and patients with type 1 diabetes (T1DM) (15.11%). Patients with T2DM had significantly higher K scores than patients with type 1. Additionally, T2DM non-insulin patients’ k-scores significantly exceeded those with T1DM. General and healthcare education both helped increase patients’ K-scores. Mostly, patients with diabetes without any complications had significantly higher knowledge compared to those having them. Lastly, regardless of whether the education was delivered by general or professional means, the effect on glycated hemoglobin (HbA1C) levels was not significant. Conclusions: Our study revealed that patients with T2DM exhibited higher knowledge than patients with T1DM. Furthermore, receiving education, whether by a healthcare professional or by general means, improved the knowledge levels of patients with T2DM but not patients with T1DM. Regarding diabetes complications, it was found that those with a higher level of knowledge had fewer complications. However, no evidence receiving education influenced the levels of HbA1C, neither in patients with T1DM nor T2DM.

## 1. Introduction

When the pancreas does not generate enough insulin or when the body cannot effectively utilize the insulin that is produced, diabetes, which is a dangerous, chronic condition, develops. Typically, raised blood glucose in uncontrolled diabetes might eventually cause significant harm to the heart, blood vessels, eyes, kidneys, nerves, and eyes [1]. Diabetes is a significant health issue; it is estimated that the prevalence of diabetes worldwide was 10.5% in 2021 and is predicted to increase to 12.2% in 2045, and it is estimated that one in every two adults with diabetes is unaware of their diagnosis. Concerning the region, the prevalence of diabetes in Saudi Arabia is 18.7%, placing it as the top fourth country in the region. Diabetes complications can compromise health and threaten life if they are not adequately treated. Acute complications play a prominent role in mortality, expense, and poor quality of life. A life-threatening outcome might result from abnormally high blood sugar if it leads to conditions like diabetic ketoacidosis (DKA) and hyperosmolar coma in both type 1 and type 2 diabetes [2]. 

Furthermore, diabetes and its complications have resulted in 6.7 million deaths, or 12.2% of the total deaths worldwide, in 2021. In addition, 20.2% of all deaths in MENA (Middle East and North Africa) are related to diabetes [1,3]. All forms of diabetes can have abnormally low blood glucose levels, which can cause seizures or unconsciousness. Additionally, managing comorbidities, besides diabetes, indirectly improves diabetic outcomes. For example, controlling blood pressure and cholesterol to lower cardiovascular risks and other consequences, as well as routine screening and early intervention for damage to the eyes, kidneys, and feet. This will subsequently improve diabetes outcomes via diet, physical exercise, and, if necessary, diabetic medication or insulin injections. The efficiency of managing diabetes ultimately depends on how well patients follow instructions and receive treatment. Therefore, patient education is crucial to managing diabetes [1]. In addition, poor education can lead to suboptimal treatment and the development of complications. For example, one of the main factors in managing DM with insulin therapy is the effectiveness of insulin techniques. A study was conducted in 2014 to determine whether proper injection technique (IT) is associated with improved glucose control over three months. The study showed that targeted, individualized training in IT is associated with improved glucose control, greater satisfaction with therapy, better and simpler injection practices, and possibly a lower consumption of insulin after only three months [4].

Furthermore, hypoglycemia symptoms and how to recognize them are a vital part of diabetes education. It is also essential to reduce impaired awareness of hypoglycemia (IAH), as it is the most critical risk factor for severe hypoglycemia [5]. In a recent study, the prevalence of IAH in patients with type 1 diabetes (T1DM) was 17%, and in patients with type 2 diabetes (T2DM) treated with insulin, it was 9.7% [6,7]. A study that was performed in Tabuk shows that the mean body mass index, waist circumference, and blood glucose levels are all reduced as a result of trained health educators providing structured diabetes self-care education to patients with diabetes [8]. Locally in Saudi Arabia, particularly in Madinah, there has not been a study on the education of diabetes focusing on its relation to glycated hemoglobin (HbA1c) as an outcome.

### Research Objectives

This study aims to evaluate patients’ knowledge of diabetes, assigning a knowledge (K) score out of 100, and investigate the possible impact of patient knowledge on diabetes control demonstrated through their HbA_1c_, as well as the presence of diabetic complications. This will be achieved by comparing the K scores based on the participants’ different characteristics and the presence of diabetic complications, as well as correlating their K scores with their HbA_1c_. 

## 2. Methods

### 2.1. Study Design and Inclusion/Exclusion Criteria

We conducted an interview-based, cross-sectional study involving adult patients with diabetes above the age of 18 years using a simple random sampling. Participants had to understand Arabic well enough to participate in the interview. Anyone below the age of 18 or who could not understand the interview language was excluded. This study was multi-center involving the largest diabetes center in Madinah (King Fahad hospital diabetes center) in addition to multiple primary health care clinics representing different geographical areas in the Madinah area (northern, eastern, western, and southern parts of the city). It lasted 15 months, between December 2022 and March 2024.

### 2.2. Sample Size Calculations

The sample size was determined using the Epi-Info program [9]. As per the General Authority for Statistics (family health survey) for diabetic adults aged 15–80 years old in Madinah, the last report was 137,205 patients (based on the latest statistics as of 2018) [10]. We considered a population size of 137,205, an alpha error of 0.05, a confidence limit of 95%, and an expected frequency of 50%. The estimated sample size was 384. Patients’ characteristics, e.g., gender and age, as well as demographic information, e.g., level of income and education, were collected and categorized according to predetermined groups.

### 2.3. Study Tool

The eligible patients were interviewed using a structured questionnaire. The Michigan Diabetes Knowledge Test (MDKT) was utilized as the main tool to assess general knowledge related to diabetes. It was developed by the Michigan Diabetes Research Center [11], which was later translated into Arabic and was tested for validity and reliability [12].

It contained three sections. Informed consent, Socio-demographic data, and the Diabetes Knowledge Test (DKT) survey, composed of 23 questions, with a focus on T1DM and T2DM to acquire diabetes patient education. No personal or identifying information was collected during this study. Patients were further stratified based on whether they attended a nutritional clinic or not and whether they received diabetic education either through general means, e.g., health leaflets, or through professional healthcare personnel, e.g., doctors or healthcare educators. The last 3 results of HbA1c levels were driven directly from the patients and not from the electronic medical records. The average of these 3 results were used to interpret the results.

### 2.4. Statistical Analysis

Data collectors were senior medical students, and data was coded, entered, and analyzed using GraphPad Prism, Version 10.2.3. Data distribution was determined using the Shapiro–Wilk test. Parametric data is presented using the mean and standard deviation; median and interquartile ranges are used for nonparametric data, while frequency and percentages are used for categorical data. Independent sample *t*-tests and Mann–Whitney U tests were used to compare 2 parametric and nonparametric groups, respectively. A one-way ANOVA was used to compare more than 2 groups with Tukey’s test for multiple comparisons, while Pearson’s correlation was used to assess the correlation between continuous variables. Results were considered statistically significant if the *p* value was less than 0.05.

### 2.5. Ethical Considerations

Ethical approval was obtained from the research ethics committee at Taibah University in Madinah (STU-22-016v2). Ethical approval was also obtained from the health affairs general directorate of the Madinah region (IRB23-033). Information was provided to all patients prior to obtaining consent. Confidentiality was also confirmed for all interviewed patients. Data is stored electronically in electronic files, and access will be granted only to the investigators of this study.

## 3. Results

### 3.1. Patients’ Characteristics

The participants in the study (N = 364) exhibited diverse characteristics. The average age was 53, with a balanced gender distribution (48.33% male, 51.67% female). Most participants were married (75.43%), and most had T2DM (48.63%) without insulin usage. Most of the studied population had income below or equal to 12,000 SAR (79.2%), with a significant percentage earning even less than 6000 SR (44.7%). On average, participants had been living with diabetes for 20 years, and educational attainment varied, with a predominant level of up to high school level (63.4%). A family history of diabetes was common, primarily in first-degree relatives (60.2%). Almost half of the patients had some sort of diabetes complication, such as retinal disease (20.8%), cardiovascular disease (12.9%), and peripheral nerve disease (9.8%). Notably, these complications were not exclusive to some patients, i.e., some patients had multiple diabetic complications. Additionally, 38.4% of participants had visited a nutrition clinic. This comprehensive profile highlights the heterogeneity within the Madinah diabetic population, emphasizing the importance of considering various factors in understanding and addressing diabetes-related issues in this community. The participants’ social and clinical characteristics are summarized in Table 1.

### 3.2. Factors Associated with Differences in Patients’ Knowledge Score

**Figure 1:**
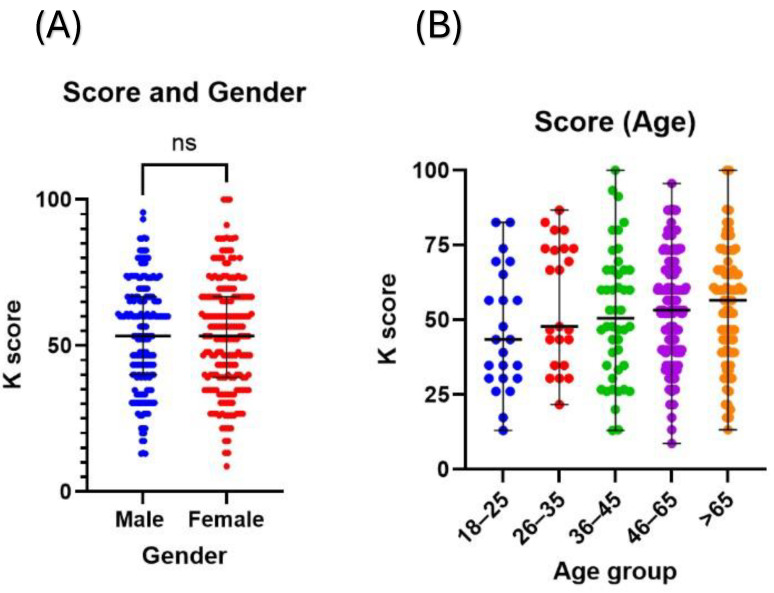

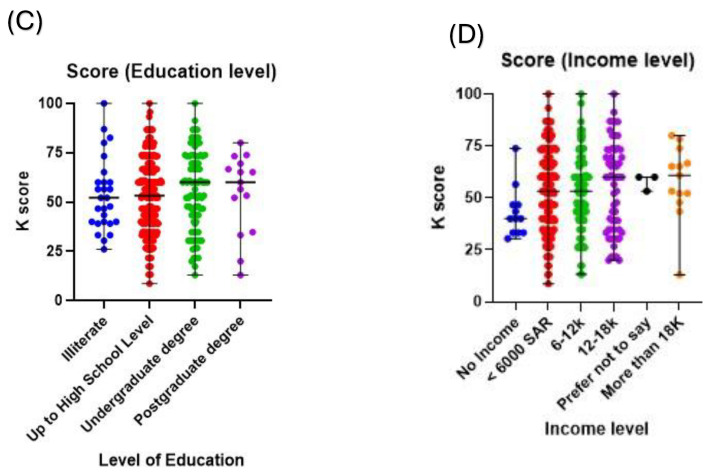
Factors influencing patients’ K scores. The figures demonstrate comparisons of K scores between patients based on their demographics, which include (**A**) gender, (**B**) age, (**C**) education, and (**D**) income level. K score: knowledge score; ns: not statistically significant.

The patients in our study, as described in the Methods section, were subjected to a questionnaire to assess their knowledge of diabetes, which gave them a knowledge (K) score out of 100. The demographic and social factors were analyzed to assess what influences patients’ K scores. Interestingly, none of the demographic characteristics, such as gender, age group, education level, or income level, significantly influenced the patients’ K scores (Figure 1A–D).

**Figure 2:**
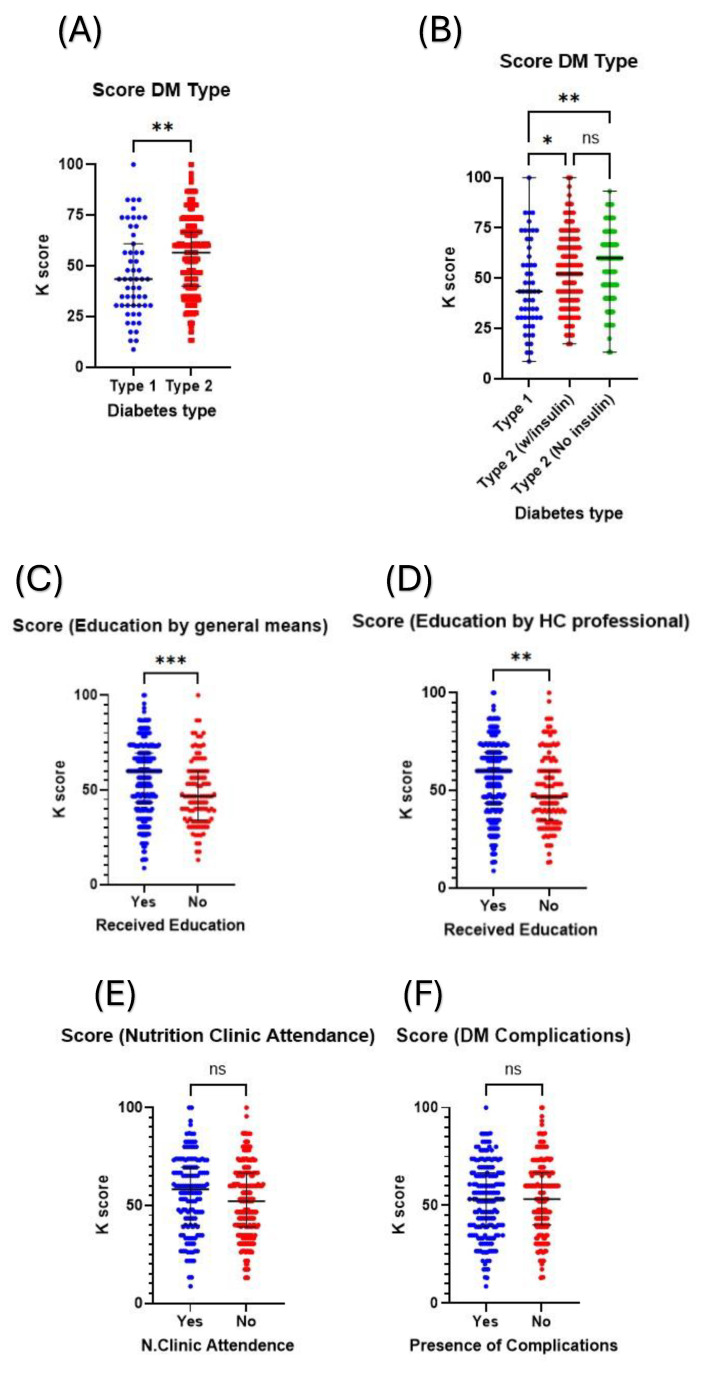
Effect of diabetes type, receiving education, and presence of complications on the patients’ K scores. The figures demonstrate comparisons of patients’ K scores (**A**) between T1DM and T2DM and (**B**) between T1DM and T2DM using and not using insulin, which show significantly higher K scores among patients with T2DM. Patients who receive education, either (**C**) by general means or (**D**) by a healthcare professional, also demonstrate higher K scores compared to those who did not. Both (**E**) the attendance at a nutrition clinic and (**F**) the presence of diabetic complications did not significantly influence the K scores. HC: Healthcare, N.Clinic: Nutrition Clinic, ns: not statistically significant, * denotes *p* value < 0.05, ** denotes *p* value < 0.01, *** denotes *p* value < 0.001.

On the other hand, the type of diabetes mellitus affected their K scores, with patients with type 2 diabetes having significantly higher K scores compared to those with type 1 (56.52 vs. 43.48; *p* value < 0.01; Figure 2A). Upon further stratification, it was found that regardless of whether insulin is used, the K scores of patients with T2DM were higher than those of patients with T1DM, but no significant difference was observed between patients with T2DM based on their use of insulin (Figure 2A,B). Factors linked to managing the patient’s condition were also assessed to determine if they influenced their K scores. These factors include complications, education about their condition through general means or healthcare professionals, and patients’ attendance at nutrition clinics. Receiving education, both by general means and by healthcare professionals, significantly improved the K scores of patients (Figure 2C,D). On the other hand, there was no statistically significant difference between those who attended nutrition clinics and those who did not (Figure 2E). Similarly, no difference in the K scores was observed between patients suffering from diabetic complications and those who did not (Figure 2F).

### 3.3. Educating Patients with Type 2 Diabetic Significantly Improves Their K Scores

**Figure 3:**
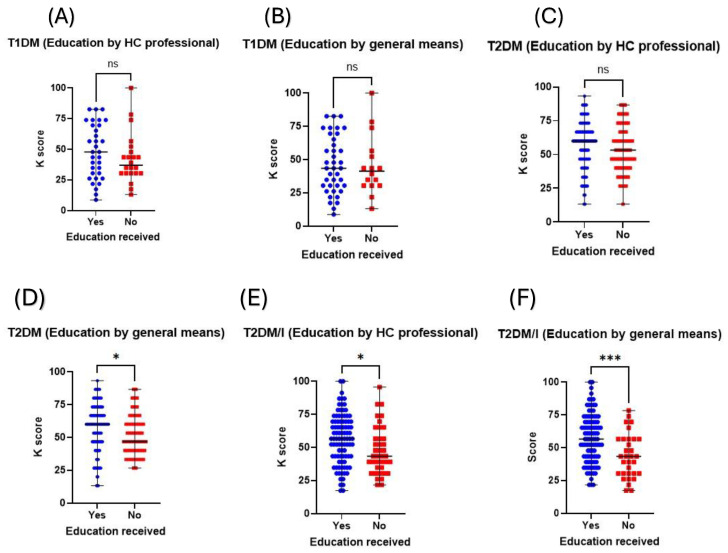
Subgroup analysis of K scores based on patients’ diabetes type and insulin use status. The figures show the comparison of K scores, based on their diabetes type and insulin use status, between those who received education and those who did not. Receiving education among patients with T1DM, either by (**A**) a healthcare professional or (**B**) general means, was not associated with any significant differences in their K scores. Patients with T2DM not on insulin had a significant difference in K score differences among those who received or did not receive education by general means (**D**), but this was not the case for (**C**) professionals. Patients with T2DM who were on insulin had significant differences in their K scores between those who received (**E**) education by a professional and (**F**) general education compared to those who did not. ns: not statistically significant, T1DM: Type 1 diabetes mellitus; T2DM: Type 2 diabetes mellitus; T2DM/I: Type 2 diabetes mellitus on insulin, * denotes *p* value < 0.05, *** denotes *p* value < 0.001.

Educating patients on their condition, whether through general means or via a healthcare professional, significantly impacted their knowledge of their condition. Further analysis was applied to evaluate whether such educational efforts influence the different subsets of patients with diabetes. Upon assessing patients with T1DM, although statistical significance was not observed, education provided by professionals influenced the K score (Figure 3A); the same observation was noticed with those who were educated by general means (Figure 3B). In this study’s cohort, patients with T2DM who did not receive insulin as part of their treatment had higher K scores when they received education from a healthcare professional than those who did not, although not statistically significant (Figure 3C). On the other hand, those who received education by general means had significantly higher K scores than those who did not (60 vs. 46.67; *p* value < 0.05; Figure 3D). The effect of education on patients’ knowledge was most pronounced in patients with T2DM who were on insulin; those who received education from a healthcare professional had significantly higher K scores than those who did not (56.52 vs. 43.48; *p* value < 0.05; Figure 3E). Additionally, patients with T2DM who were on insulin and who received education by general means had significantly higher K scores than those who did not (56.52 vs. 43.48; *p* value < 0.001; Figure 3F).

### 3.4. Educating Patients with Diabetes Influences Their Diabetic Control

**Figure 4:**
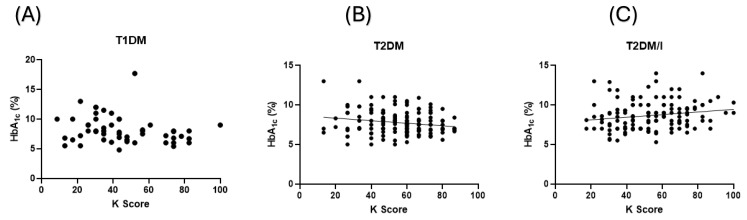
The association between the K scores and the levels of HgA_1c_. The figures demonstrate (**A**) a lack of correlations between patients with T1DM K scores and their levels of HgA_1c_, in contrast to (**B**) patients with T2DM, who showed a significant negative correlation (*p* value < 0.05) between their K scores and HgA_1c_ levels. (**C**) Patients with T2DM on insulin demonstrated a significant positive correlation (*p* value < 0.05) between their K scores and HgA_1c_ levels. The regression lines included in (**B**,**C**) reflect the direction of the correlations between K score and HbA_1c_. HbA_1c_: Hemoglobin A_1c_, K score: knowledge score, T1DM: Type 1 Diabetes Mellitus, T2DM: Type 2 Diabetes Mellitus, T2DM/I: Type 2 Diabetes Mellitus on Insulin.

Interestingly, patients’ levels of hemoglobin A1c were not equally affected by education, as demonstrated in their K scores, that they may or may not receive for their conditions. Patients with T1DM did not demonstrate any correlations between their K scores and their hemoglobin A1c levels (*p* value > 0.05; Figure 4A). In contrast, patients with T2DM seemed to benefit from their education, as their K scores negatively correlated with their hemoglobin A1c levels (*p* value < 0.05; Figure 4B). Lastly, patients with T2DM on insulin had a positive correlation between their K scores and their hemoglobin A1c levels (*p* value < 0.05; Figure 4C).

### 3.5. Educating Patients with Diabetes Improves Their Quality of Life by Reducing Diabetic Complications

**Figure 5:**
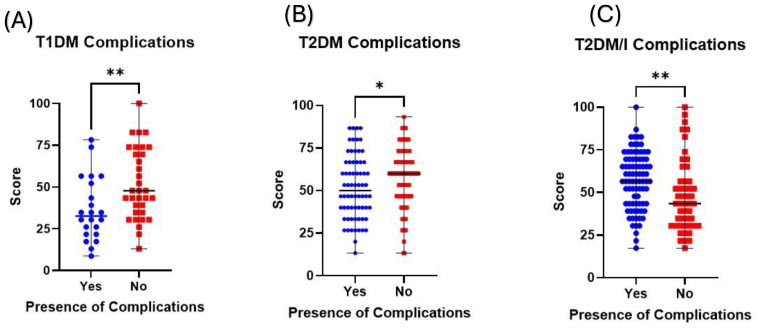
The impact of patients’ K scores on the presence or absence of diabetic complications. The dot plots compare the K scores among those with and without diabetic complications among (**A**) patients with T1DM, (**B**) patients with T2DM, and (**C**) patients with T2DM who are on insulin. * Denotes a *p* value < 0.05, ** Denotes a *p* value < 0.01.

Educating patients, whether through professional means or others, increases their knowledge about the condition and enhances their involvement in managing their diabetes. So, the better involvement and management of their cases will be reflected in their quality of life by reducing diabetic complications, such as diabetic foot. Evidently, in this study, patients with T1DM who did not suffer any complications of diabetes had significantly higher K scores than those with complications (47.83 vs. 32.61; *p* value < 0.01; Figure 5A). Similarly, patients with T2DM without diabetic complications had significantly higher K scores compared to those with complications (60 vs. 50; *p* value < 0.05; Figure 5B). Interestingly, patients with T2DM who were using insulin and who suffered from diabetic complications had significantly higher scores compared to those without complications (56.52 vs. 43.48; *p* value < 0.01; Figure 5C).

The course of disease may better explain such counterintuitive results. Patients with uncontrolled T2DM who are receiving maximum doses of antidiabetic agents will receive insulin to control their glycemic levels. Hence, this population is more likely to develop diabetic complications due to their uncontrolled glycemic levels.

## 4. Discussion

This study aimed to evaluate patients’ knowledge of diabetes, assigning a knowledge (K) score out of 100. Demographic factors such as gender, age group, education level, and income level did not significantly impact patients’ knowledge scores. This could be attributed to the similarity of the background of the studied population since the majority had a considerably low or very low-income status, and the majority had lower than graduate-level education. However, the type of diabetes had a significant effect, with patients with T2DM demonstrating higher knowledge scores compared to patients with T1DM, which could be attributed to the duration of diabetes that is usually longer in T2DM and the maturity of the older age population of patients with T2DM. Further analysis within the T2DM group revealed that patients not on insulin had significantly higher knowledge scores than those with T1DM. One theory to explain this could be that on top of patients with T2DM being more mature, those with higher K scores are under better control and do not require insulin therapy.

Additionally, factors related to managing the condition were examined, including complications, education through general means or healthcare professionals, and attendance at nutrition clinics. Patients who received an education by general means and healthcare professionals showed significantly improved knowledge scores compared to patients who did not receive it. Contrary to our study that showed a lack of significant influence from demographic factors (suggesting that irrespective of gender, age, education, or income, patients had similar levels of knowledge about diabetes), a study performed in Jordan found a better knowledge score in patients younger than 45 [13].

On the other hand, another study performed on the population of Thailand by Phoosuwan et al. revealed a lack of significance between patients’ age and their overall knowledge score [14]. Among diverse demographic groups, the significant difference in knowledge scores between patients with T2DM and T1DM was the nature of their diabetes condition itself, which plays a role in patients’ understanding. Further stratification revealing higher scores in patients with type 2, not on insulin, suggests that treatment methods may also impact knowledge levels. Nevertheless, it is worth saying that it was expected to find higher K scores in the population with T1DM rather than what we have found in our study because it was expected that with the younger generation tending to have easier accessibility to the internet, sources of information, and health care facilities that this would positively impact their K score. Only 15% of the studied population had T1DM. This could also reflect certain patients with T1DM who are not reflective of the entire population with T1DM since some of the population with T1DM tend to follow up more with specialized endocrinology clinics rather than in primary health care centers or diabetes centers.

A study by Ruszkiewicz et al. revealed that patients with T1DM had higher knowledge scores compared to T2DM [15]. The lack of a significant difference between patients who attended nutrition clinics and those who did not suggests that these clinics might not be a primary source of knowledge improvement for patients in this context. Alternatively, there are not enough structured education programs in these clinics at the public primary health care centers. In this study, patients with T1DM who did not suffer any complications from diabetes had significantly higher K scores than those with complications (47.83 vs. 32.61; *p* value < 0.01; Figure 4A). Similarly, patients with T2DM without diabetic complications had significantly higher K scores compared to those with complications (60 vs. 50; *p* value < 0.05; Figure 4B). This demonstrates the importance of education and knowledge on reducing diabetes complications. This is supported by a study performed on the population of Thailand by Phoosuwan et al., who reported that patients with higher knowledge scores were less likely to experience complications related to diabetes [14].

Our study revealed a significant positive association between receiving any form of education (general or professional) and higher knowledge scores in patients with T2DM, both with and without insulin use. Notably, the group with T2DM without insulin use exhibited higher K scores when receiving only general education. However, our study did not find a statistically significant difference between patients with T1DM who received an education in any form (general or professional) or those who did not receive an education. The findings of our study align with recent studies demonstrating an increased K score (using the same K measuring tool) in patients with T2DM, including insulin users, after the application of targeted education programs like diabetes self-management education (DSME) compared to control cases [16,17]. Another systematic review of education programs for patients with T2DM provided by different health care providers (nurse, pharmacist, and DM educator) in the Middle East indicated significant improvement in knowledge scores in three review studies. However, heterogeneity in assessment tools limited conclusive interpretations [18]. In addition, a systematic review of RCTs on pharmacist-led education programs for T1DM and T2DM suggested that pharmacist-led self-management interventions improve diabetes knowledge [19]. In the review by the King’s Fund on informal and flexible approaches to self-management education, the report concluded the need for diverse educational options for individuals with diabetes. It recommended further research into level two initiatives. Level 2 education programs generally involve educating patients with variable tools like face-to-face groups, technology, and internet-based approaches. The findings of this review could explain the result of our research on T2DM, which received a higher score when receiving general education compared to no education at all [20]. In the case of patients with T1DM, a study was conducted in Birmingham, UK, which used a 12-month education program for patients who visited a diabetes clinic at Dudley Road Hospital. This study concluded that follow-up of the study group after 12 months showed significant improvement in knowledge scores [21]. Similarly, a Turkish study with 524 patients with diabetes concluded that higher levels of general knowledge and diabetes complications may be attributed to the impact of education provided both by professionals and by general means [22]. Contrary to our study, which did not find significant improvement in knowledge, these studies found significant improvement in the knowledge score, which could be explained by the presence of a structured education program, longer follow-up, and larger sample size. Education programs and other methods of educating patients with diabetes led to increased knowledge in patients with diabetes about their condition and helped them better understand the obstacles in optimizing the management provided for them. As shown in our research, general means of education are not to be neglected due to their effect and reach in some patient groups. Further research is needed to assess and construct education programs for patients with T1DM. Diabetes complications are major determinants of disease activity and controllability, and they often contribute to impaired quality of life in patients with diabetes. Patients’ level of knowledge about diabetes helps them achieve better control of the disease, therefore reducing the risk of complications related to the course of the disease. In our study, both patients with T1DM and T2DM with better knowledge were less likely to have complications of diabetes than those whose knowledge was worse. Similar findings were reported multiple times in different studies. For example, in a study done on the population of China by Qiu et al. 2020, it was found that knowledge about diabetes in patients with T2DM significantly reduced the risk of complications [23]. Furthermore, another study on 100 patients with T2DM found that better knowledge reduces the likelihood of diabetes-related complications [24]. Locally in Saudi Arabia, evidence on the association between knowledge about diabetes and the risk of developing complications is lacking. A compelling finding that illuminates the complex relationship between insulin therapy, disease progression, and diabetic complications is the observed phenomenon in which patients with T2DM who were using insulin and experienced diabetic complications had significantly higher scores compared to those without complications. From our perspective, this could be because the duration of the illness could be a significant factor associated with complications; thus, patients with complications are more likely to have longstanding diabetes, which reflects the knowledge they have gained throughout their medical journey and frequent clinic visits may also be contributing factors. Also, this might highlight an important gap between the knowledge and attitude of patients based on their acquired knowledge. Finally, we think that higher scores in patients with complications might be due to the aftermath, meaning that this category of patients might have started seeking more knowledge after they have started to suffer serious complications; this should be explored in future studies. An article published in 2003 found that individuals who were on insulin had significant knowledge gaps about diabetes [25]. The absence of a difference in knowledge scores between patients with and without complications indicates that the presence of complications does not correlate only with higher or lower diabetes knowledge but rather depends on their attitude based on the acquired knowledge. Compared to a study performed in the population of Thailand by Phoosuwan et al., patients with higher knowledge scores are less likely to experience diabetes-related complications [14]. The correlation that has been shown between the use of insulin, diabetic problems, and higher scores emphasizes the difficulty of controlling T2DM and the demand for an all-encompassing approach to treatment. This entails managing and preventing diabetic complications, addressing other modifiable risk factors, and maximizing glycemic control. More research is required to improve the outcomes for individuals with T2DM and to gain a better understanding of the mechanisms behind this connection. Not to forget that developing complications is not only on the patient’s hands; this could reflect the failure of the treating physician or the system handling chronic illnesses and improper follow-up and management plans. The results, which indicate that patient education did not considerably affect HbA1c levels, emphasize the need for a more comprehensive comprehension of the variables affecting diabetes care. Since education is only one part of the equation, to enhance glycemic control and outcomes, future prospective research should concentrate on finding efficient methods for providing customized, all-encompassing education interventions that address the various requirements and difficulties faced by individuals with diabetes and other confounding factors and controlling them as much as possible.

### Limitations

The sample size was not completely met (364/385 participants) (94.5%) because of the difficulty of the interview-based questionnaire. Most of the studied population came from a low socioeconomic status background, making the generalizability of the study findings difficult. The last three HbA1c readings were obtained from participants and not from electronic records. Other contributing factors that could affect HbA1c levels were not considered, such as CBC. The study’s cross-sectional nature limits the ability to establish a causal relationship between diabetes education and observed outcomes. Longitudinal studies or randomized controlled trials in the future would provide more robust evidence for the impact of education on diabetes complications and control. Future studies should carefully address other diabetic comorbidities [26], which would significantly improve the recommendations for future public health policymakers [27].

## 5. Conclusions

In conclusion, we found that even though the demographic factors of the individuals did not influence their knowledge, the type of diabetes was significant in terms of differences between the groups, with patients with T2DM having better knowledge than patients with T1DM. Furthermore, this study found that receiving education, whether by a healthcare professional or by general means, improved the knowledge levels of patients with T2DM but did not influence the knowledge levels of patients with T1DM. Diabetes complications were found in this study to be related to the level of knowledge, with those with a higher level of knowledge having fewer complications. However, in terms of HbA1C levels, it was found that receiving education did not influence the levels of HbA1C, neither in patients with T1DM nor T2DM, which could be for other confounding factors in this population.

## Figures and Tables

**Table 1:** The characteristics of the study participants.

Social and Clinical Characteristics	
N	364
Age	53 ± 13 (15–80)
Gender (%)	
Male	176 (48.33%)
Female	188 (51.67%)
Marital status (%)	
Single	34 (9.38%)
Married	275 (75.43%)
Divorced	16 (4.41%)
Window/er	39 (10.78%)
Diabetes type and treatment (%)	
Type 1	55 (15.11%)
Type 2 using insulin	132 (36.26%)
Type 2 not using insulin	177 (48.63%)
Family income (%)	
No income	12 (3.3%)
Less than 6000 SR	163 (44.7%)
6000–12,000 SR	114 (31.2%)
12,000–18,000 SR	59 (16.4%)
More than 18,000 SR	13 (3.65)
Does not want to answer	3 (0.8%)
Duration of diabetes in years (range)	20 ± 12 (1–50)
Level of education	
Illiterate	27 (7.4%)
Up to high school degree	230 (63.4%)
Undergraduate degree	92 (25.1%)
Postgraduate degree	15 (4.1%)
Family history of diabetes (%)	
1st degree relative (Mother, father, sister, brother, son, daughter)	219 (60.2%)
2nd degree relative (grandmother, grandfather, uncle, aunt)	23 (6.3%)
Both 1st and 2nd degree relative	35 (9.6%)
I do not Know	39 (10.6%)
No family history of diabetes	48 (13.3%)
Complications of diabetes (%)	
Retinal disease	76 (20.8%)
Cardiovascular disease	47 (12.9%)
Diabetic foot	4 (1.1%)
Peripheral nerve disease	36 (9.8%)
Renal disease	11 (3.1%)
I do not have any complications of diabetes	190 (52.3%)
Visited Nutrition Clinic (%)	
Yes	140 (38.4%)
No	224 (61.6%)

## Data Availability

The raw data supporting the conclusions of this article will be made available by the authors on request.
